# Spontoneous Evolution

**Published:** 1847-08

**Authors:** 


4. *Spontaneous Evolution.*—At a meeting of the Obstetrical Society of Edinburgh, at which a discussion followed the narration of some cases of spontaneous cephalic and pelvic evolution, Dr. Simpson gave the following general deductions respecting the process. He observed—1st. That spontaneous evolution in transverse presentations was not so rare as some authors averred, and that it would probably occur oftener if timely assistance were not profferred. 2d. That under some circumstances arm and shoulder cases should probably be left to be expelled by the mechanism of spontaneous evolution, assisting, if necessary, this mechanism by art. 3d. That this ought to be our practice if, in an arm or shoulder case, the chest and trunk of the child be already thrust down into the cavity of the pelvis; for to turn under such a complication, and with that object, attempt to push back the body of the child into the cavity of the contracted uterus, would risk a rupture of its coats. 4th. That if the process of spontaneous evolution fails, two operations have been recommended to effect delivery, viz.: evisceration and decapitation. 5th. That evisceration is only applicable to cases of *pelvic* spontaneous evolution demanding operative interference; and decapitation is only applicable to *cephalic* spontaneous evolution. 6th. That in all common transverse presentations, seen before the body and bulk of the infant is doubled and thrust into the cavity of the pelvis *turning* is the proper practice. 7th. That a child of common size would never, in a transverse presentation, be thrust into the cavity of the pelvis, unless the pelvis were large in its dimensions; and hence, when the process of spontaneous evolution is found in an advanced stage, it is almost a certain sign that the pelvis is of such a size as to give a chance of its completion.—*Month. Jour.*, *ibid.*

